# Synthetic Cannabinoid AB-FUBINACA Negatively Impacted the Male Fertility and Induced Testicular Toxicity

**DOI:** 10.1007/s11419-025-00739-y

**Published:** 2025-10-13

**Authors:** Ayman Alzu’bi, Ejlal Abu-El-Rub, Fatimah A. Almahasneh, Rawan Almazari, Amani Kasasbeh, Heba F. AI-jariri, Amneh Alrabie, Raed M. Al-Zoubi

**Affiliations:** 1 Department of Basic Medical Sciences, Faculty of Medicine, Yarmouk University, Irbid, 211-63 Jordan; 2 Surgical Research Section, Department of Surgery, Hamad Medical Corporation, Doha, Qatar; 3 Department of Biomedical Sciences, QU-Health, College of Health Sciences, Qatar University, 2713 Doha, Qatar; 4 Department of Chemistry, Jordan University of Science and Technology, P.O.Box 3030, Irbid, 22110 Jordan

**Keywords:** Synthetic cannabinoids, AB-FUBINACA, Male fertility, Oxidative stress, Apoptosis

## Abstract

**Purpose:**

The recreational use of synthetic cannabinoids (SCs) by adolescents and adults has markedly increased in recent years. Previous studies demonstrated that exposure to SCs is associated with multiple adverse health effects. Nevertheless, little is known about the effects of these substances on male fertility. The current study aimed to investigate the toxicological effects of subacute exposure to synthetic cannabinoid AB-FUBINACA on male reproductive system in mice.

**Methods:**

Adult male Balb/c mice received daily intraperitoneal injections of various doses of AB-FUBINACA (0.75, 1.5, and 3 mg/kg for 3 weeks). Using biochemical and molecular methodologies, the impact of AB-FUBINACA on serum levels of reproductive hormones, sperm viability as well as various parameters in testicular tissue were evaluated.

**Results:**

Our findings demonstrated that AB-FUBINACA induces dose-dependent reduction in testosterone levels in the serum, but not in follicle-stimulating hormone or luteinizing hormone. AB-FUBINACA treatment also causes a significant dose related decrease in sperm viability. These findings were associated with higher level of oxidative stress (GP91 expression and malondialdehyde level) and elevated expression of key regulators of apoptosis (Bax and caspase-3) as well as reduced expression of mitochondrial respiratory chain complexes SDHB (II), UQCRC2 (III), and ATP5a (V) in the testicular tissue.

**Conclusion:**

From these findings, it can be concluded that exposure to AB-FUBINACA can interfere with the normal physiology and functioning of the male reproductive organs. Hence, gaining insight into the mechanisms by which SCs interfere with male fertility could guide future interventions and treatments.

## Introduction

Synthetic cannabinoids (SCs) are a category of designer substances that target the endocannabinoid system (ECS) to imitate the biological effect of tetrahydrocannabinol (THC), [1, 2]. Compared to THC, the majority of SCs demonstrate significantly greater binding affinities to the cannabinoid receptors 1 and 2 (CB1 and CB2) [3, 4]. In addition, it was shown that these substances also interacted with non-cannabinoid targets, which could result in distinct pharmacologic effects [5, 6]. Most SCs are classified under the category of schedule I drugs in USA, making their possession and use illegal; nevertheless, the recreational use of these substances among young people has markedly increased over the past two decades [7]. The use of SCs has been connected to wide range of adverse health effects, including agitation, anxiety, nausea, seizures, hallucinations, paranoid behavior, depressed breathing, and cardiac complications [8]. The increased use of these substances among adolescents and young adults has raised concerns about potential negative impact on male fertility [8, 9].

Research suggests that the ECS is a critical modulator for male reproduction at multiple stages of the hypothalamus-pituitary–gonadal (HPG) axis [10, 11]. It has been shown that the key components of ECS, endocannabinoids (e,g., anandamide and 2-archidonoylglycerol) and cannabinoid receptors, are present in the hypothalamus, pituitary gland, and testicular tissue, including Sertoli and Leydig cells as well as germ cells [12, 13]. Evidence indicates that activation of cannabinoid receptors in the hypothalamus and pituitary gland can potentially affect the secretion of follicle-stimulating hormone (FSH) and luteinizing hormone (LH) that are essential to reproductive functions [14]. Furthermore, the activation of cannabinoid receptors in the testes has been demonstrated to be involved in localized functions, such as testosterone secretion and spermatogenesis [14, 15]. It is therefore possible that disturbing the delicate balance of the ECS by THC and other synthetic cannabinoid receptor agonists can pose significant risks to male reproductive health [16].

The link between exogenous manipulation of ECS and potential adverse impact on male reproductive health has been observed in both human and animal studies. Human studies have revealed that exposure to cannabis is strongly associated with reductions in sperm count, concentration, and motility [17–20]. Recreational cannabis use also appears to be associated with altered DNA integrity and considerable poor morphology in sperm [19, 21, 22]. Furthermore, despite being inconclusive, studies of hormonal changes suggest that cannabis consumption can be related to disruption in the plasma levels of testosterone, FSH, and LH [21, 23, 24]. These effects were also replicated in animal studies, where exposure to cannabis or THC was also shown to have potential effect on sperm parameters and hormonal balance [25–27]. On the other hand, information on the impact SCs use on male reproduction are still very limited in literature. Chronic use of HU210, a synthetic analog of THC, was found to be associated with impaired spermatogenesis and reduced sperm motility [28]. Administration of synthetic cannabinoid JWH-018 in male rats induced various degenerative changes in testes and reduced sperm count and motility [9]. Given the structural uniqueness of each type of SCs as well as the much higher affinity and potency for most SCs to bind to cannabinoid receptors compared to THC, additional studies are necessary to explore the unexpected adverse effects of these drugs on male reproduction and elucidate the underlying mechanisms for these effects.

AB-FUBINACA is a widely abused third generation synthetic cannabinoid [29]. Forensic investigations have linked this drug to numerous hospitalizations and deaths [30, 31]. We have recently shown that its chronic administration in mice induces memory impairment and hippocampal neurotoxicity [32] and its acute administration is associated with nephrotoxic side effects [33]. It has been suggested that SCs induce oxidative stress, inflammation, and mitochondrial dysfunction as underlying mechanisms for SCs related to toxic effects on multiple organs [8]. In this study, using male mouse animal model, we evaluated the effect of administration of various doses of this drug on sperm number and viability and the plasma levels of testosterone, FSH, and LH. In addition, its effects on various parameters in testes, including oxidative stress, inflammation, apoptosis, and mitochondrial dysfunction were also investigated.

## Material and methods

### Chemicals

AB-FUBINACA (Cayman Chemical, Item No. 14039). enzyme-linked immunosorbent assay (ELISA) kits: Mouse Testosterone ELISA Kit (ELK Biotechnology, Catalog No. ELK10970), Mouse Luteinizing Hormone ELISA Kit (ELK Biotechnology, Catalog No. ELK2368), and Mouse Follicle Stimulating Hormone ELISA Kit (ELK Biotechnology, Catalog No. ELK4808). thiobarbituric acid reactive substances assay kit (R&D Systems, KGE013). primary antibodies for CB1 (Santa Cruz Biotechnology, sc-293419), GP91 (Santa Cruz Biotechnology, sc-74514), NOS3 (Santa Cruz Biotechnology, sc-376751), Bax (sc-20067), Caspase-3 (sc-56053), NF-κB (Santa Cruz Biotechnology, sc-166588), NDUFB8 (Abcam, ab192878), SDHB (Santa Cruz Biotechnology, sc-271548), UQCRC2 (Santa Cruz Biotechnology, sc-390378), COX5a (Santa Cruz Biotechnology, sc-376907), and ATP5A (Santa Cruz Biotechnology, sc-136178). secondary antibodies; anti-mouse HRP conjugated secondary antibody (BioRad Cat # 1706516) and anti-rabbit HRP conjugated secondary antibody (BioRad Cat # 1706515).

### Animals and treatments

All animal procedures were conducted in compliance with the ethical guidelines approved by the Animal Care and Use Committee at Yarmouk University (Protocol number IACUC/2021/14). Adult male Balb/c mice, aged 10 weeks and weighing 23–25 g, were used in this study. mice were distributed into four groups (*n* = 5 per group). AB-FUBINACA was dissolved in a vehicle (5% ethanol, 5% Tween 80, and 90% saline). Mice in the experimental groups received daily intraperitoneal injections of AB-FUBINACA at doses of 0.75 mg/kg, 1.5 mg/kg, or 3 mg/kg for three weeks. The dose range of AB-FUBINACA was determined based on previous studies [31–33]. Mice in the control group were administered the vehicle for the same duration. All animals were sacrificed 24 h after the last injection by cervical dislocation. Both testes and epididymides were collected for analysis.

### Assessment of epididymal sperm viability and sex hormones levels

The cauda epididymis was dissected and gently squeezed with forceps to release spermatozoa into a Petri dish containing 200 μL of pre-warmed phosphate-buffered saline. Sperm viability was evaluated using the trypan blue exclusion test. A 50 μL drop of the sperm suspension was taken and mixed with an equal volume of 0.4% trypan blue, and viable sperm percentages were determined using the Corning® CytoSmart Cell Counter.

Serum levels of reproductive hormones (testosterone, LH and FSH) were quantified (24 h after the last injection) using ELISA kits, following the manufacturer’s protocols. The assays were based on competitive inhibition immunoassay principles.

### Assessment of malondialdehyde (MDA) levels

The concentration of MDA in testicular tissue were determined to assess lipid peroxidation using commercially available thiobarbituric acid reactive substances assay kit. The assay procedure was carried out according to the manufacturer’s protocol. Absorbance of the MDA–TBA adduct was measured spectrophotometrically at 532 nm. MDA concentrations were expressed as nmol/mg of protein.

### Western blotting protein analysis

Protein expression levels of CB1, GP91, NOS3, Bax, Caspase-3, NF-κB, and mitochondrial complexes in the testicular tissue were evaluated using western blotting. Briefly, total protein was extracted from the testicular tissues using RIPA lysis buffer and being homogenized using the sonicator. Protein levels were measured using NanoDrop™ Lite spectrophotometer (ThermoFisher Scientific). A total amount of 50 μg of protein was loaded onto SDS–PAGE gels. Following electrophoresis, proteins were transferred to PVDF membrane and incubated with appropriate primary (1:500) and secondary antibodies (1:2000). The membranes were visualized using VILBER FUSION Gel Documentation System (Vilber, France), and protein bands were quantified using ImageJ for densitometry.

### Co-immunoprecipitation

Co-Immunoprecipitation (Co-IP) was carried out following the protocol provided by Santa Cruz Biotechnology. Total protein lysates were prepared by being extracted from the testicular tissues of each group using RIPA lysis buffer and homogenized using the sonicator. The lysates were precleared using a preclearing matrix. To form the IP antibody–matrix complex, 40–50 μL of suspended (25% v/v) IP matrix and 1–5 μg of antibody (CB1 antibody) were incubated in 500 μL PBS overnight at 4 °C. Subsequently, 300 μg of total protein was added to the pelleted matrix and incubated overnight at 4 °C. The immunoprecipitated proteins were then analyzed by SDS-PAGE and western blotting, as described above. The developed PVDF membrane was incubated with p-Ser/Phosphoserine primary antibody (Cat # sc-81514) to detect the phosphorylation level of CB1.

### Statistical analysis

The statistical analyses were carried out utilizing GraphPad Prism (version 8.0.0 for Windows, GraphPad Software, CA, USA). The data underwent analysis through one-way analysis of variance (ANOVA) followed by Tukey's test. The findings were displayed as mean ± standard error of the mean (SEM). A *p* value of less than 0.05 was considered a significant difference compared to the vehicle group.

## Results

### Effects of AB-FUBINACA treatment on reproductive hormones

**Fig. 1:**
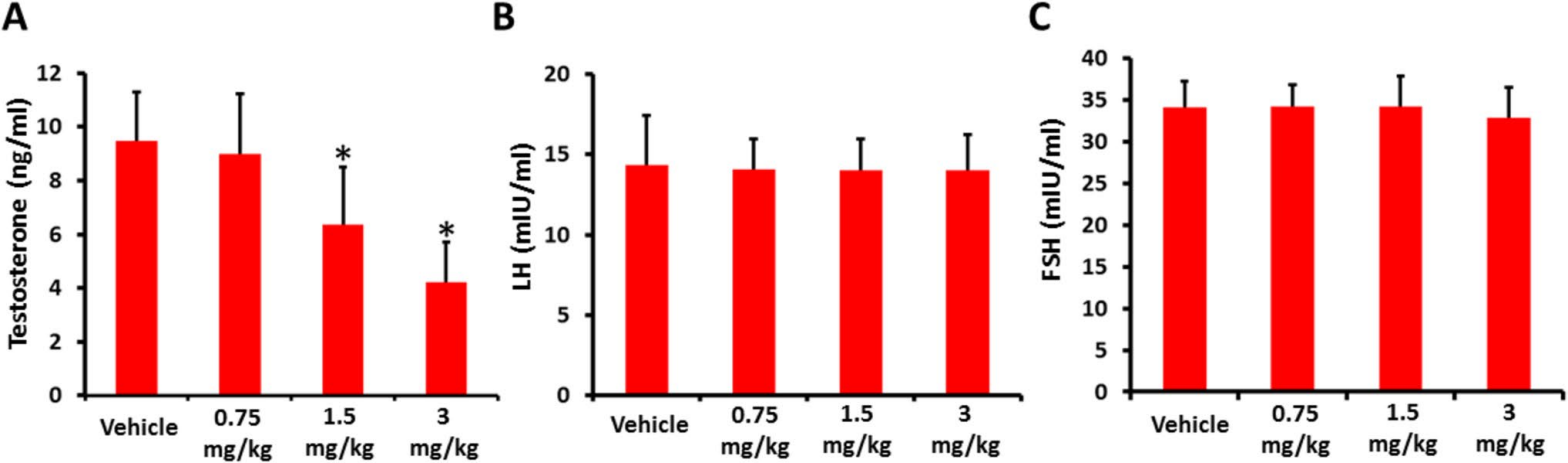
The serum levels of testosterone (**A**), luteinizing hormone (LH, **B**), and follicle-stimulating hormone (FSH, **C**) in vehicle and AB-FUBINACA treated groups detected by ELISA assays. Data presented as mean ± SEM (*n* = 5); *P < 0.05 is statistically significant compared to vehicle group

The effects of AB-FUBINACA treatment on the serum levels of testosterone, LH and FSH were evaluated. Our results showed that the serum testosterone levels are negatively influenced by AB-FUBINACA treatment. While there was no statistically significant difference between 0.75 mg/kg AB-FUBINACA treatment and vehicle group, 1.5 and 3 mg/kg AB-FUBINACA treatments resulted in a significant reduction in the serum testosterone levels compared to vehicle group (Fig. 1A). Conversely, the serum LH and FSH levels did not statistically differ for any treatment and vehicle groups (Fig. 1B,C).

### Effects of AB-FUBINACA treatment on sperm viability

**Fig. 2:**
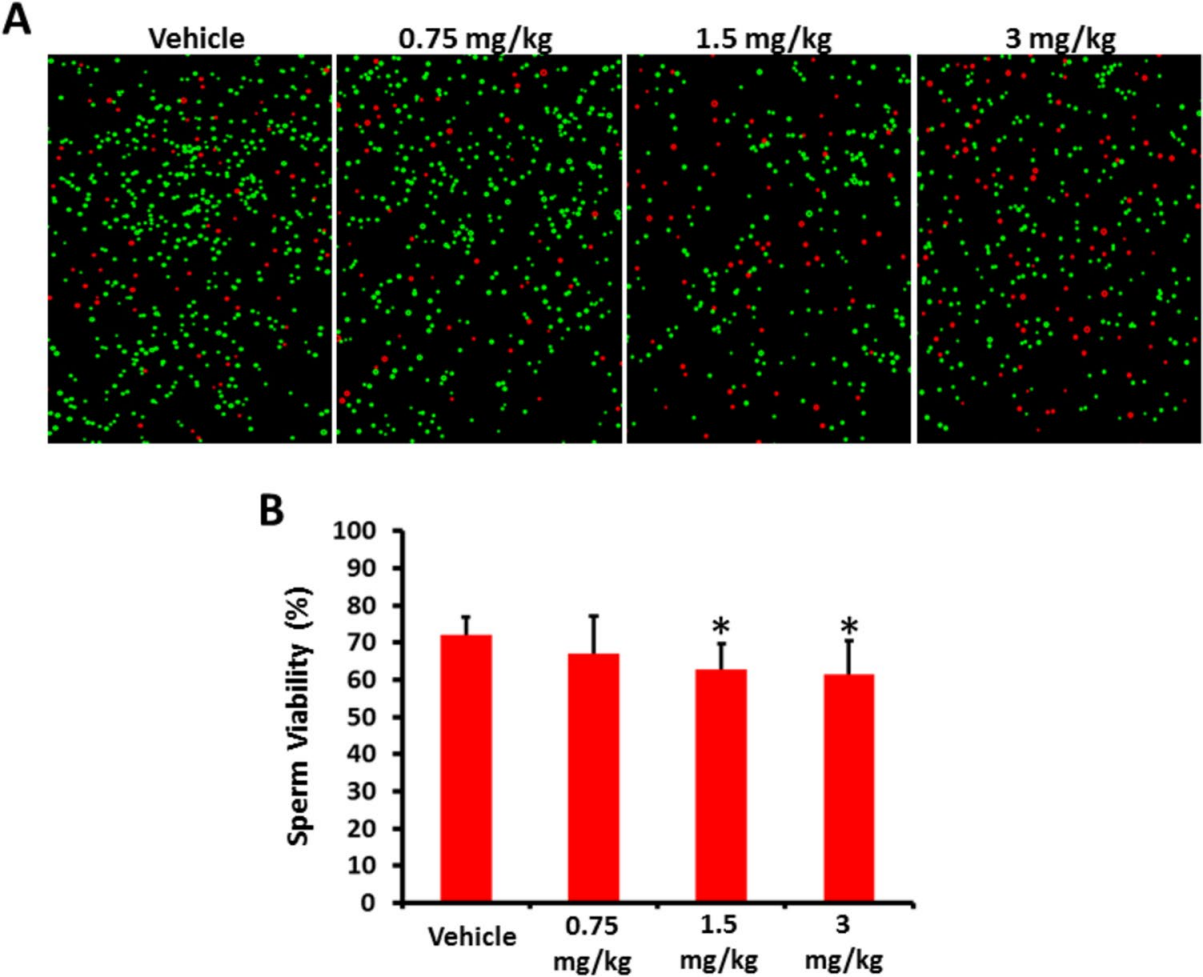
**A** Representative images of sperm viability in vehicle and AB-FUBINACA treated groups determined by the trypan blue exclusion test; live sperms (green) and dead sperms (red). **B** The percentage of viable sperm in each group. Data presented as mean ± SEM (*n* = 5); *P < 0.05 is statistically significant compared to vehicle group

Our results showed that 1.5 and 3 mg/kg AB-FUBINACA treatments caused a significant reduction in the proportion of live sperm compared to vehicle group. In addition, significant reduction in sperm viability was observed these two treatment groups and 0.75 mg/kg AB-FUBINACA treatment group (Fig. 2).

### Effects of AB-FUBINACA treatment on CB1 receptor expression in testis

**Fig. 3:**
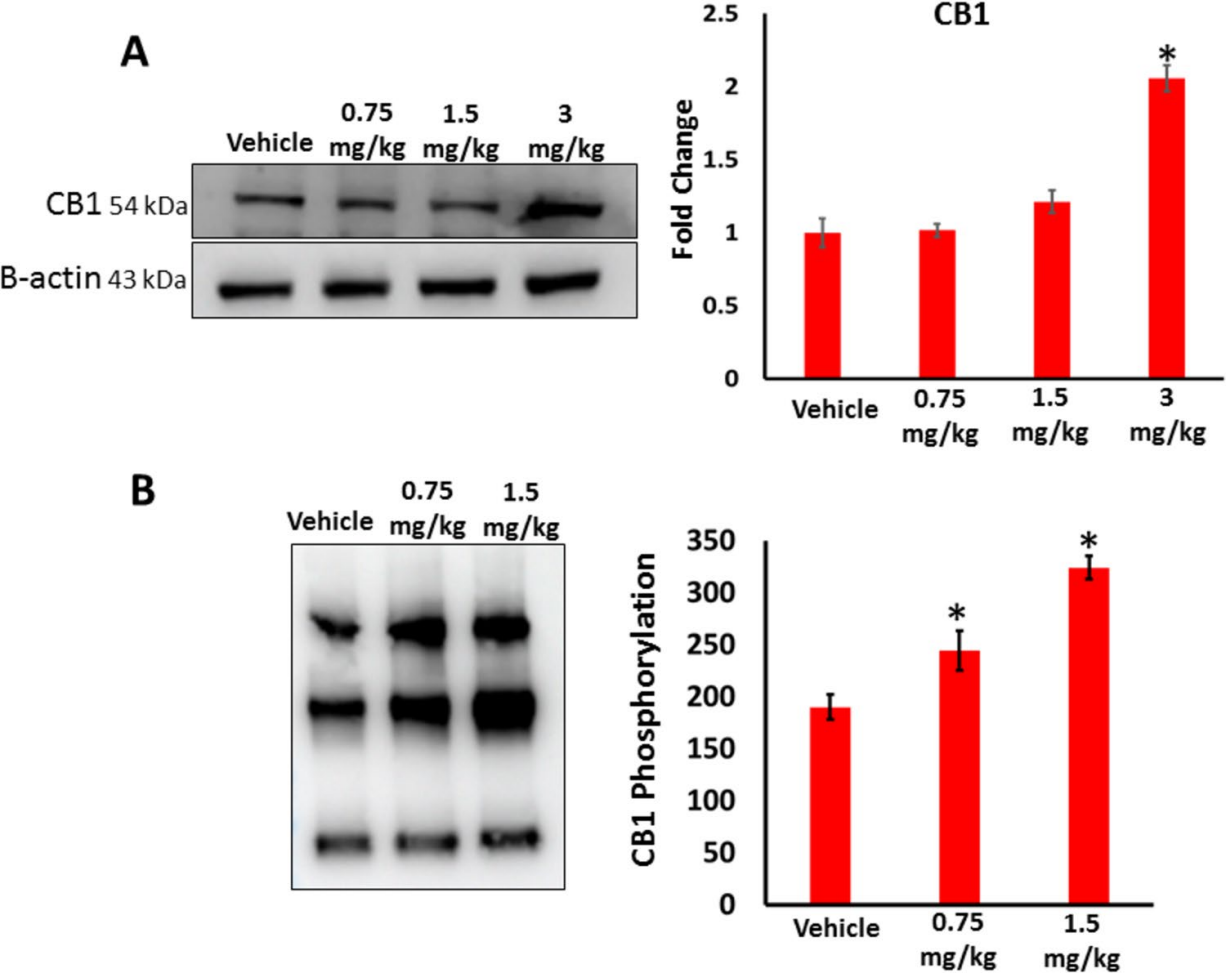
**A** The expression of CB1 receptor in the testes of vehicle and AB-FUBINACA treated groups detected by western blot analysis. **B** The phosphorylation of CB1 receptor in the testes of vehicle and AB-FUBINACA treated groups detected by Co-immunoprecipitation assay. Data presented as mean ± SEM (*n* = 3); *P < 0.05 is statistically significant compared to vehicle group

We assessed the effect of AB-FUBINACA treatment on CB1 receptor expression and activation in testes. Our western blotting analysis revealed that CB1 receptor expression was significantly higher in 3 mg/kg AB-FUBINICA treatment compared to the vehicle group (Fig. 3A). Post-translational phosphorylation is considered one of the major modifications that increases the expression and activity of many proteins. Thus, we also performed co-immunoprecipitation (Co-IP) assay to explore the effect of AB-FUBINICA in enhancing the phosphorylation of CB1 receptor. The results revealed that the phosphorylation of CB1 receptor significantly increased with increasing dose of AB-FUBINACA compared to the vehicle group (Fig. 3B). This indicates that AB-FUBINICA induces activation of CB1 receptor by phosphorylation.

### Effects of AB-FUBINACA treatment on the level of oxidative stress and apoptosis in the testes

**Fig. 4:**
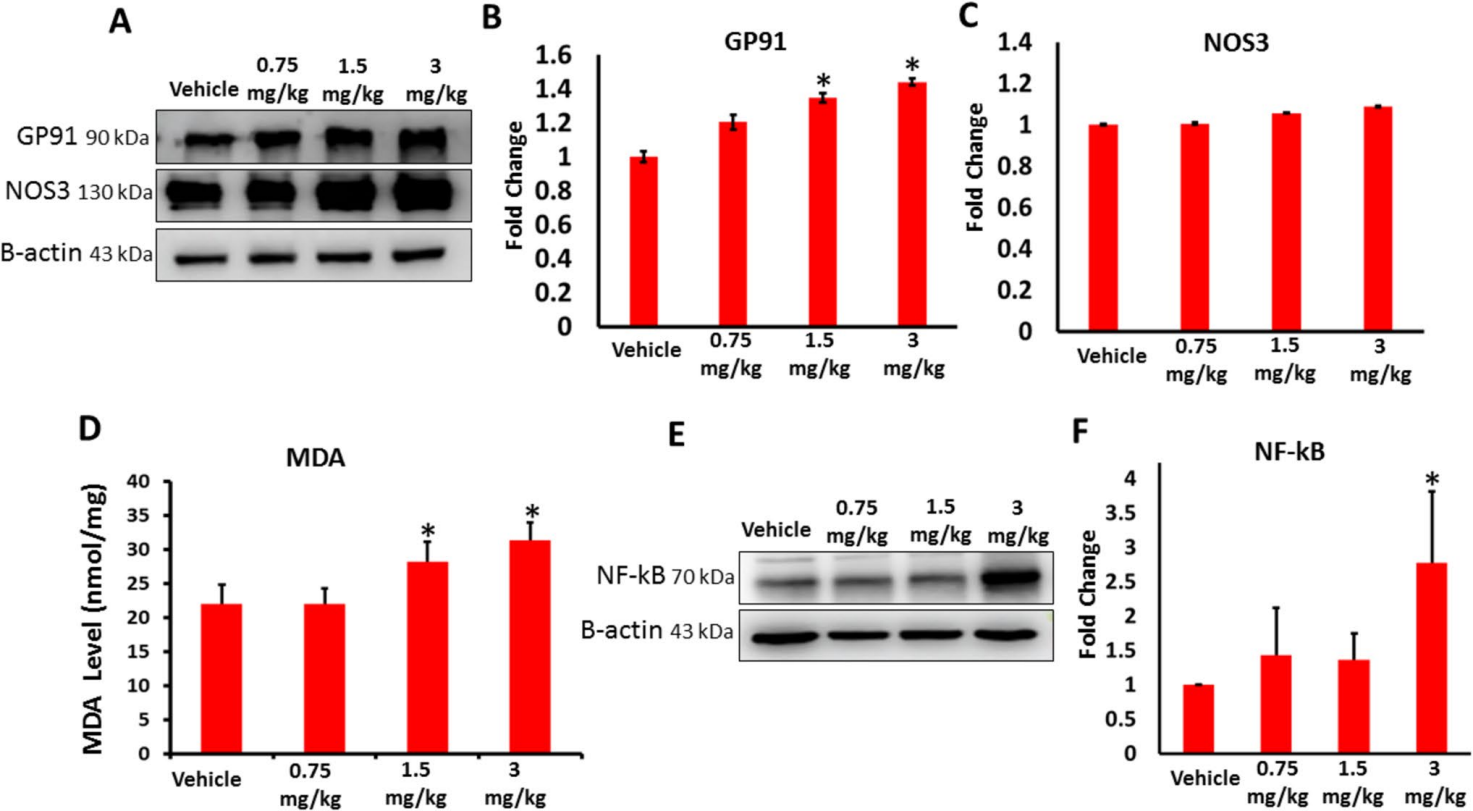
**A**–**C** The expression of GP91 and NOS3 in the testes of vehicle and AB-FUBINACA treated groups detected by western blot analysis. **D** The serum level of malondialdehyde in the testes of vehicle and AB-FUBINACA treated groups determined by TBARS assay (MDA). **E**, **F** The expression of NF-kB in the testes of vehicle and AB-FUBINACA treated groups detected by western blot analysis. Data presented as mean ± SEM (*n* = 3); *P < 0.05 is statistically significant compared to vehicle group

The impact of AB-FUBINACA on oxidative stress levels in testicular tissue was examined by assessing the protein expression of GP91 (NOX2) and NOS3 and quantifying the level of lipid peroxidation as assessed by measuring the level of MDA. Our western blot analysis revealed significant increase in the expression of NOX2 was in 1.5 and 3 mg/kg AB-FUBINACA treatments compared to vehicle group (Fig. 4A,B). Although there was trend for increased expression of NOS3 in AB-FUBINACA treatment groups compared to vehicle group, this increase was not statistically significant (Fig. 4A,C). However, the evidence for increased oxidative stress in the testicular tissue was confirmed by significant increase of MDA level observed in 1.5 and 3 mg/kg AB-FUBINACA treatments compared to vehicle group (Fig. 4D). Since this potential increased oxidative stress level can trigger inflammatory response in the testicular tissue, we tested the expression of Nuclear Factor Kappa B (NF-kB), a protein complex that is essential for the initiation of the of inflammatory responses, and the finding revealed a significant increase in the expression of NF-kB in 3 mg/kg AB-FUBINACA treatment, but not in 0.75 nor in 1.5 mg/kg AB-FUBINACA treatments, compared to vehicle group (Fig. 4E,F).

**Fig. 5:**
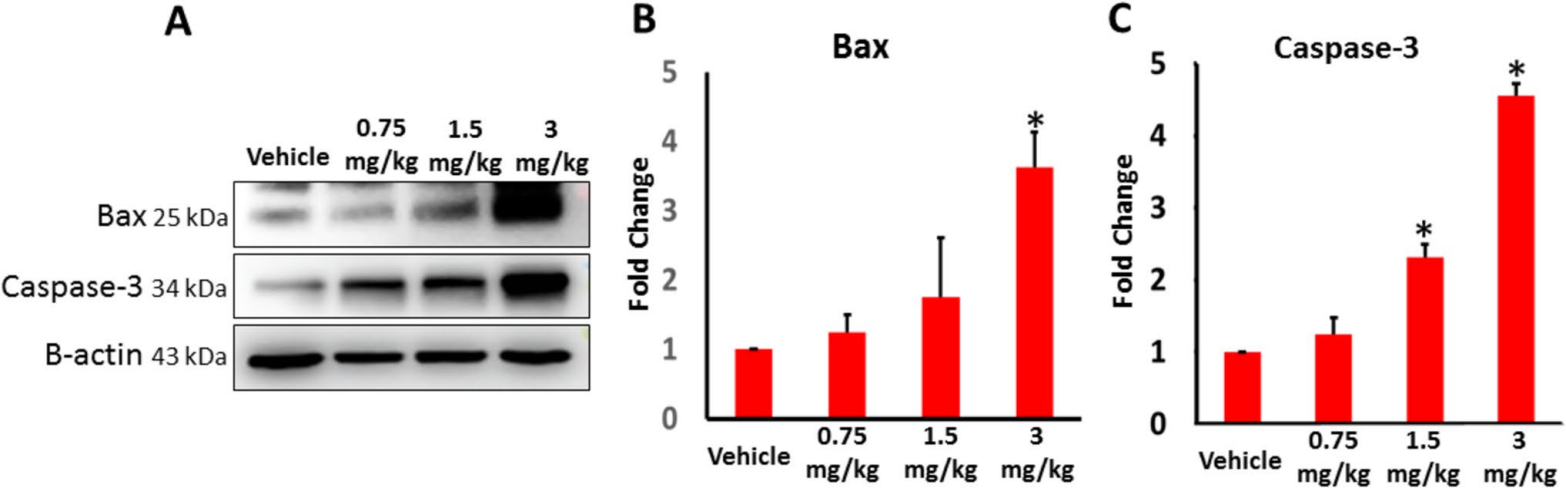
The expression of Bax and Caspase-3 in the testes of vehicle and AB-FUBINACA treated groups detected by western blot analysis. Data presented as mean ± SEM (*n* = 3); *P < 0.05 is statistically significant compared to vehicle group

In order to evaluate if AB-FUBINACA treatment can also induce apoptosis in the testicular tissue, we measured the expression levels of pro-apoptotic marker Bax and a key zymogen in cell apoptosis Caspase-3. As seen in Fig. 5A–C, Bax expression was significantly higher in 3 mg/kg AB-FUBINACA treatment, and caspase-3 expression was shown to be significantly higher in both 1.5 and 3 mg/kg AB-FUBINACA treatments compared to vehicle group, indicating potential emergence of irreversible apoptosis in the testicular tissue.

### Effects of AB-FUBINACA treatment on the mitochondrial respiratory chain complexes in the testes

**Fig. 6:**
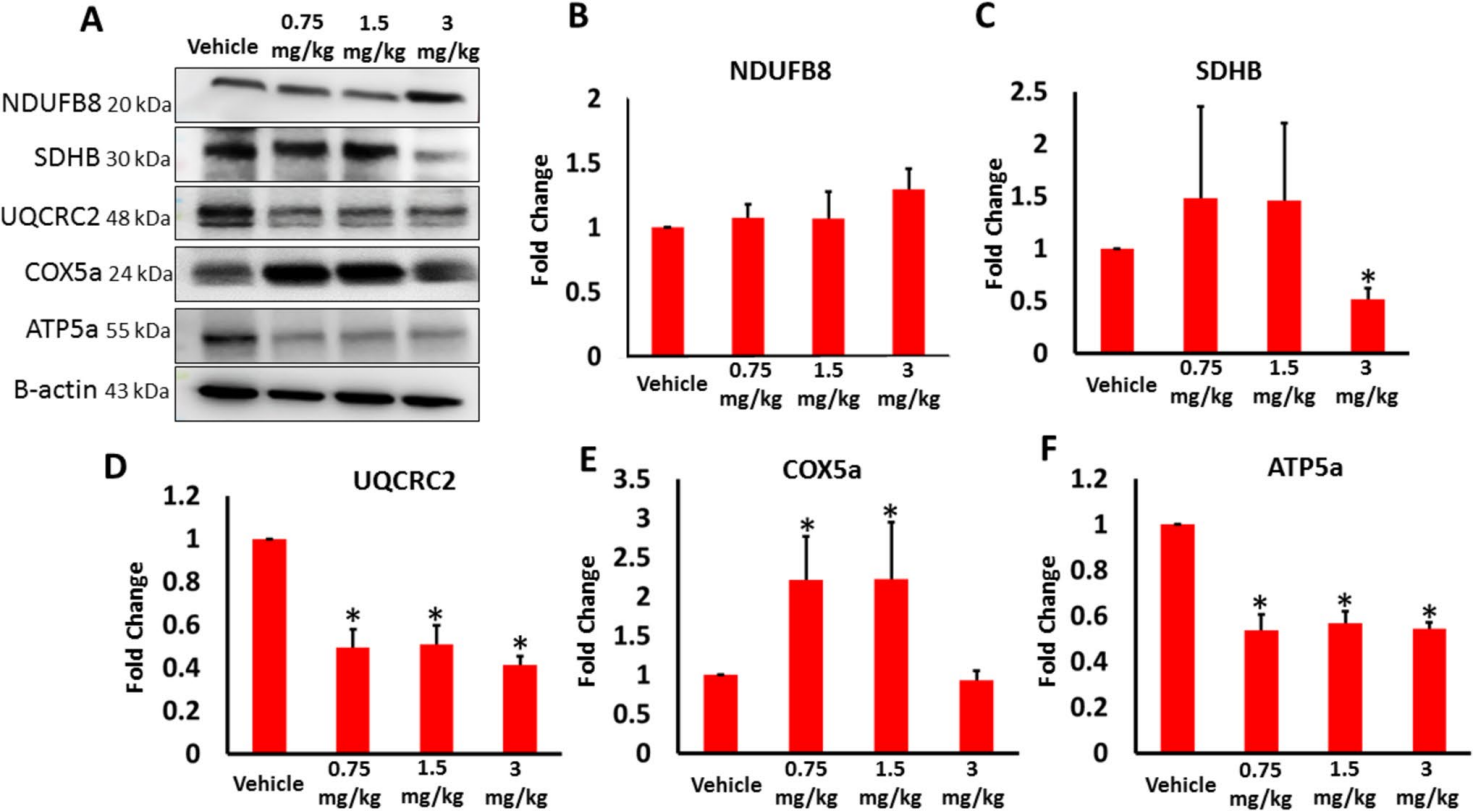
The expression of mitochondrial respiratory chain complexes I (NDUFB8), II (SDHB), III (UQCRC2), IV (COX5a), and V (ATP5a) in the testes of vehicle and AB-FUBINACA treated groups detected by western blot analysis. Data presented as mean ± SEM (*n* = 3); *P < 0.05 is statistically significant compared to vehicle group

The high level of Bax intrigued us to further assess the effect of AB-FUBINICA on the mitochondrial dynamics. Evidence presented demonstrate that cannabinoids mediate their pharmacological effects by modulating the intramitochondrial signaling and respiration through activation of CB1 receptors found on mitochondria (mtCB1) [34]. To explore if AB-FUBINACA treatments influence the mitochondrial function in testicular tissue, we evaluated the expression of mitochondrial complexes I–V (Fig. 6). Our findings revealed that AB-FUBINACA treatments particularly caused a significant reduction in the expression of SDHB (II), UQCRC2 (III), and ATP5a (V) indicating an impairment in their activity. SDHB expression was significantly decreased in 3 mg/kg AB-FUBINACA treatment, whereas UQCRC2 and ATP5a expressions were significantly decreased in the three AB-FUBINACA treatments compared to vehicle group (Fig. 6C, D and F).

## Discussion

Synthetic cannabinoids have started being widely consumed by men of reproductive age. Prior research has shown that the exposure to exogenous cannabinoids can interrupt the normal physiology and functioning of the male reproductive organs and negatively impact male fertility [17, 20]. Nevertheless, the underlying mechanisms by which these substances interfere with male reproductive health remains not fully investigated. Therefore, gaining insights into these mechanisms is of paramount importance to guide future interventions and treatments.

In this study, we demonstrated that AB-FUBINACA induces dose-dependent reduction of serum level of testosterone. In contrast, the levels of LH and FSH were not significantly changed by AB-FUBINACA treatment. Although it has been suggested that cannabinoids may have an indirect impact on testis functions through the HPG axis [14, 15], the reported effect of cannabinoids on serum testosterone, LH and FSH levels is still widely variable across current studies [20]. While previous studies indicated that exposure to cannabinoids can be associated with depressed serum levels of LH and/or testosterone and minimal to no effect on FSH level [17, 35, 36], other studies reported insignificant role of exogenous cannabinoids in disturbing HPG axis [18, 37, 38]. Depending on our data, we suggest that AB-FUBINACA may have localized effects in the testes which can interfere with testosterone secretion.

Compatible with reduced testosterone level, we observed that AB-FUBINACA treatment also causes a significant decrease in sperm viability. This result is consistent with human and animal studies showed that exposure to cannabinoids leads to negative consequences on semen quality [17–20]. The negative effect of AB-FUBINACA on testosterone level and sperm viability may be explained as a result of concomitant alteration in the redox status and the activity of mitochondrial complexes in the testicular tissue (discussed below) which can adversely affect the testicular histology and the process of spermatogenesis [9].

The findings of this study demonstrated that AB-FUBINACA treatment was linked to a dose-dependent increase in the expression of oxidative stress markers and elevated levels of MDA in the testes, indicating enhanced oxidative stress. Moreover, the treatment significantly upregulated the expression of NF-κB, a key regulator of the expression of many other pro-inflammatory genes [39]. These results are consistent with our previous reports, which showed that AB-FUBINACA administration induced oxidative stress and inflammation in the kidney and hippocampus of treated mice [32, 33]. The current findings also align with in vitro studies examining the neurotoxic effects of SCs, where oxidative stress and inflammation were identified as central mechanisms underlying SC-induced toxicity in SH-SY5Y neuronal cells [40, 41]. The role of endocannabinoid system (ECs) in modulating cellular redox homeostasis is well-documented. Excessive activation of ECs, particularly via CB1 receptor stimulation, has been linked to increased oxidative stress and pro-inflammatory responses in multiple tissue types [42–46]. Notably, CB1 activation in human macrophages has been shown to promote the production of reactive oxygen species (ROS) and TNF-α cytokine, both of which were partially suppressed by pharmacological inhibition of CB1 [47]. In line with these observations, our data showed that AB-FUBINACA treatment enhanced both the expression and phosphorylation of CB1 receptors in testicular tissue (Fig. 3), suggesting a potential mechanistic link between CB1 activation and the observed oxidative and inflammatory responses seen in AB-FUBINACA treated mice as they are logical consequence to CB1 receptor activation. Additionally, our research revealed that treatment with AB-FUBINACA increases the expression of key regulators of apoptosis, specifically Bax and caspase-3, within the testicular tissue. While apoptosis can be directly associated with the modulated oxidative-inflammatory cascade, activation of CB1 receptor was also demonstrated to trigger ROS-independent activation of the mitogen-activated protein kinase pathway, resulting in cell death [48]. Collectively, the findings of this study highlight the key role of oxidative stress, inflammation, and apoptosis in SCs-induced testicular toxicity and as an underlying mechanism of various degenerative changes observed in the testes, such as seminiferous tubule degeneration, upon exposure to SCs [9, 28].

Finally, this study indicated that AB-FUBINACA treatment can be associated with a significant reduction in the activity of mitochondrial complexes II (SDHB), III (UQCRC2), and V (ATP5a) in the testes. Indeed, extensive body of evidence for the toxicological effects of THC and SCs seems to be mainly related to mitochondrial dysfunction [34]. In both vitro and vivo conditions, direct activation of CB1 receptor by exogenous cannabinoids was associated with changes in integrated mitochondrial function in several cell types [49–52]. Exposure to cannabinoids was also shown to induce inhibition of respiratory chain complexes in brain and heart mitochondria [53, 54]. Moreover, we have previously shown that AB-FUBINACA treatment reduce the expression of mitochondrial complexes I (NDUFB8), III (UQCRC2), and IV (COX5a) in the renal tissue [33]. This impairment in the mitochondrial respiratory enzyme activity can be a trigger of many cellular process, such generation of ROS and activation of caspase-dependent cell death by apoptosis [34]. Thus, it can be conceded that the potential reduction in the activity of mitochondrial complexes II, III, and V is a key inciter for increased oxidative stress and apoptosis observed in the testes of FUBINACA treated mice.

## Conclusion

This study demonstrates that AB-FUBINACA, a synthetic cannabinoid, exerts dose-dependent adverse effects on male reproductive health in mice. The administration of AB-FUBINACA resulted in a notable decrease in serum testosterone levels and sperm viability without altering LH or FSH levels, indicating a direct testicular toxic effect. The compound also induced oxidative stress, inflammation, and apoptosis in the testes marked by increased expression of oxidative stress markers (NOX2, MDA), pro-inflammatory mediator NF-κB, and apoptotic proteins Bax and caspase-3. These deleterious effects appear to be mediated by the upregulation and activation of the CB1 receptor and subsequent impairment of mitochondrial respiratory chain complexes II, III, and V. Collectively, these findings highlight the potential reproductive toxicity of AB-FUBINACA and underscore the need for conducting intensive research to apprehend the long-term impacts and the dose cut-off for observable adverse effects of other synthetic cannabinoids on male fertility.

## Data Availability

The data that supports the findings in this study are available from the corresponding authors upon reasonable request.
